# Longitudinal Study on Methicillin-Resistant *Staphylococcus pseudintermedius* in Households

**DOI:** 10.1371/journal.pone.0027788

**Published:** 2011-11-23

**Authors:** Laura M. Laarhoven, Phebe de Heus, Jeanine van Luijn, Birgitta Duim, Jaap A. Wagenaar, Engeline van Duijkeren

**Affiliations:** 1 Department of Infectious Diseases and Immunology, Faculty of Veterinary Medicine, Utrecht University, Utrecht, The Netherlands; 2 Central Veterinary Institute of Wageningen UR, Lelystad, The Netherlands; 3 Laboratory for Zoonoses and Environmental Microbiology, National Institute of Public Health and the Environment, Bilthoven, The Netherlands; University of Iowa, United States of America

## Abstract

Methicillin-resistant *Staphylococcus pseudintermedius* (MRSP) is an emerging pathogen in dogs and has been found in Europe, Asia and North America. To date most studies are one-point prevalence studies and therefore little is known about the dynamics of MRSP in dogs and their surrounding. In this longitudinal study MRSP colonization in dogs and the transmission of MRSP to humans, contact animals and the environment was investigated. Sixteen dogs with a recent clinical MRSP infection were included. The index dogs, contact animals, owners and environments were sampled once a month for six months. Samples taken from the nose, perineum and infection site (if present) of the index cases and contact animals, and the nares of the owners were cultured using pre-enrichment. Index cases were found positive for prolonged periods of time, in two cases during all six samplings. In five of the 12 households that were sampled during six months, the index case was intermittently found MRSP-positive. Contact animals and the environment were also found MRSP-positive, most often in combination with a MRSP-positive index dog. In four households positive environmental samples were found while no animals or humans were MRSP-positive, indicating survival of MRSP in the environment for prolonged periods of time. Genotyping revealed that generally similar or indistinguishable MRSP isolates were found in patients, contact animals and environmental samples within the same household. Within two households, however, genetically distinct MRSP isolates were found. These results show that veterinarians should stay alert with (former) MRSP patients, even after repeated MRSP-negative cultures or after the disappearance of the clinical infection. There is a considerable risk of transmission of MRSP to animals in close contact with MRSP patients. Humans were rarely MRSP-positive and never tested MRSP-positive more than once suggesting occasional contamination or rapid elimination of colonization of the owners.

## Introduction

Methicillin-resistant *Staphylococcus pseudintermedius* (MRSP) has recently emerged as a significant pathogen in companion animals [1]. Most infections caused by MRSP are skin infections such as pyoderma. Other infections such as otitis externa, (surgical) wound infections and urinary tract infections can also be associated with MRSP [1]–[4]. The predominant clone circulating in Europe with sequence type (ST) 71 often contains genes that confer resistance to multiple antimicrobials routinely used in small animal practice [5]. Human infections with MRSP have been described; however, this is very uncommon [6]–[9]. The prevalence of MRSP has recently been studied in various dog populations [2], [10]–[12]. Rates vary widely among dogs in the community, 1.5%–4.5%, and among dogs at veterinary clinics, 2.1%–30% [10], [11], [13], [14], [15]. These cross-sectional studies have shown that MRSP is distributed worldwide. However, these studies only provide information from a single sampling. Little is known about the persistence of MRSP in dogs and their surrounding, including the humans and animals in close contact with the MRSP patient. It is often unclear if dogs or humans are actually colonized persistently or transiently or merely contaminated with MRSP. Investigations into long-term colonization with MRSP in dogs and humans are lacking, but are essential for the differentiation between short-term and long-term colonization and for a better understanding of the transmission of MRSP and the subsequent development of infection control measurements. The objectives of this study were to evaluate longitudinally MRSP colonization in dogs and to study the transmission to humans, contact animals and the environment.

## Materials and Methods

### Study design

Dogs with a recent clinical MRSP infection, which had been diagnosed at the Veterinary Microbiological Diagnostic Centre (VMDC), the Netherlands, between September 2009 and January 2010, were included in the study. During this period 27 patients had been identified at the VMDC and the owners were contacted after permission from their veterinarian. Sixteen (59%) owners agreed that their dogs, contact animals and the household members could be included. The main reason for owners to deny participation was that their veterinarian was not willing to participate in the study. Since March 2010, within seven months of the initial diagnosis of MRSP infection, the index cases, contact animals, owners and environment were sampled once a month for six months. Sampling was approved by the Medical Ethical Committee of Utrecht University (METC 09-399/C) and the Experimental Animal Committee (DEC 2009.II.10.093). All participants completed a written informed consent.

### Sampling

Nasal and perineum swabs were taken each month from the index case and contact animals using a sterile cotton-wool swab (Cultiplast ®). If the index case had clinical signs of an infection, an additional swab was taken from the site of infection (e.g., the vertical ear canal or a skin lesion).

In addition, nasal swabs were taken from the owners and other household members.

In each household, three samples from the environment were taken each month using moist wipes (Sodibox, s1 kit Ringer's solution, France). These environmental wipes were taken from the sleeping place of the index case, the feeding place and one site not physically accessible to the animals, i.e. above a door or on a cabinet. A surface of approximately 20×20 cm was sampled. Each wipe was taken wearing new sterile gloves to prevent cross-contamination. First and last samples were taken by the researcher. The other samples were taken by the owners or the veterinarian and sent to the laboratory.

### Microbiological analysis and genotyping

The swabs and wipes were analyzed individually using a pre-enrichment containing Mueller Hinton broth with 6.5% sodium chloride [16]. After overnight incubation at 37°C, 1 ml of the pre-enrichment was transferred into 9 ml selective enrichment of phenyl red mannitol broth with 75 mg/L aztreonam and 5 mg/L ceftizoxime (bioMérieux, Marcy-'l Etoile, France). After overnight incubation at 37°C, 10 µl of the selective enrichment broth was inoculated onto sheep blood agar (Biotrading, The Netherlands). Suspected colonies were identified as members of the *Staphylococcus intermedius* group (SIG) using standard techniques including colony morphology, tests for catalase, coagulase and API ID32 Staph (bioMérieux). *S. pseudintermedius* isolates were identified using PCR-restriction fragment length polymorphism (RFLP) assay based on the *Mbo*I-digestion pattern of a PCR-amplified internal fragment of the *pta* gene as described [17]. In addition, isolates were tested for the *mecA* gene [18]. The index dogs and contact animals were classified as MRSP-positive when one or more samples from the animal were MRSP-positive.

From each household the first and last MRSP isolates from the index case, the contact animal, the owner and the environment, if present, were genotyped. The MRSP isolates were typed with multilocus sequence typing (MLST), pulsed-field gel electrophoresis (PFGE), *spa* typing and SCC*mec* typing as previously described [19]-[24]. MLST targeting four genes: *agrD*, *cpn60*, *pta* and *tuf* was performed. The allele numbers and sequence types (ST) were assigned by comparison to allele sequences present in the NCBI nucleotide database and using the key table for MLST typing of *Staphylococcus intermedius* group (SIG) strains [19]. All novel allele sequences were assigned by the MLST database curator [5]. PFGE was performed using *Sma*I and *Cfr*9I digestion. PFGE was run for 24h at 5.6V/cm and with pulsed time ramping from 2 to 5 s [22]. *Spa* typing was performed according to described protocols [21], [24], using the primers SPspaF (5′-AAGTAGTGATATTCTTGCT-3′) and SPspaR (5′-CCAGGTTGAACGACATGCAT-3′). For determination of the SCC*mec* elements, the SCC*mec* type II/III was detected with the primers described by Descloux et al. [23] and all other SCC*mec* types were detected with the multiplex assays described by Kondo et al. [20].

## Results

### Index cases

The 16 index dogs had pyoderma (n = 5), otitis externa (n = 5), post-operative wound infections (n = 4), non-surgical wound (n = 1) and rhinitis (n = 1). Two index dogs were sampled only once, because one of them was euthanized and the owner of the other dog did not longer want to participate in the study. Two index cases were sampled only three or five times respectively, because in the first case the dog had no longer clinical signs of infection and was repeatedly MRSP-negative and in the second case the owner went on a holiday for several months.

A total of 229 swabs were taken from the index dogs, of which 61 (26.6%) were found MRSP-positive (Table 1). The prevalence of MRSP in the index dogs from the first to the sixth sampling was 87.5% (14/16), 71.4% (10/14), 42.9% (6/14), 46.2% (6/13), 30.8% (4/13) and 58.3% (7/12) respectively. Of the 12 index dogs, that were sampled for six months, two dogs were continuously MRSP-positive, five dogs were intermittently MRSP-positive, four dogs became MRSP-negative during the six months and one dog was never found MRSP-positive after the initial MRSP-positive sample (Table S1). One dog (household 1) was found MRSP-positive more than one year after the initial sample. In 10 of the 12 dogs the clinical signs persisted during the study period of six months. One dog occasionally showed clinical signs and one dog did not show clinical signs during six months. The MRSP-positive sites of an index dog showed considerable variation during the samplings (Table S1).

**Table 1:** Number of MRSP+ samples found at the different sampling sites.

	Number of samples	MRSP+ samples (%)	MRSP+ site	MRSP+ samples per site (%)
**Index dogs**	229	61 (26,2)	Nose	31 (21,3)
			Perineum	29 (47,5)
			Infection site	19 (31,2)
**Contact animals**	68	13 (19,1)	Nose	7 (53,8)
			Perineum	5 (38,5)
			Infection site	1 (7,7)
**Humans**	140	5 (3,57)	Nose	5
**Environment**	236	43 (18,2)	Feeding place	18 (41,9)
			Sleeping place	18 (41,9)
			Inaccessible place	7 (16,2)
**Total**	673	122 (18,1)		

MRSP was found on swabs from the perineum (n = 29), the infection site (n = 19) and the nose (n = 13) (Table 1).

### Contact animals

Seven contact animals, six dogs and one cat, from seven households were included in the study. In six of these seven households MRSP-positive contact animals were found (Table S1). A total of 68 swabs were taken from the contact animals of which 13 (19.1%) were found MRSP-positive. The prevalence of MRSP in the contact animals from the first to the sixth sampling was 71.4% (5/7), 40.0% (2/5), 0% (0/5), 0% (0/5), 20% (1/5) and 50% (2/4), respectively. Generally, MRSP-positive contact animals were only found in combination with MRSP-positive index dogs. However, in one household the index dog became MRSP-negative while the contact animal was repeatedly MRSP-positive. In one household (household 16) the contact animal showed signs of an ear infection and was also sampled at the infection site in addition to the samples from nose and perineum. MRSP was cultured from swabs taken from the nose (n = 7), the perineum (n = 5) and on one of the swabs taken from the infection site of the contact animal in household 16 (Table 1).

### Humans

Twenty-five persons living in the same household as the index dogs were included in the study. A total of 140 nasal swabs were taken of which five (3.6%) were found MRSP-positive (Table 1). In the first sampling, 3/25 (12.0%) humans from three different households were MRSP-positive. During the following four samplings no human nasal samples were MRSP- positive. In the last sampling 2/22 (10.0%) humans from the same household were MRSP-positive (Table S1). In this household the clinical condition of the index case had worsened and MRSP was also found in the index dog, the contact dog and the environment. After testing MRSP-positive, two of the five owners were re-tested repeatedly during the study period and none of the owners were tested MRSP-positive more than once. The other three owners were not re-tested, because in one household the index dog was euthanatized and in the other household the two owners were tested MRSP-positive only in the last sampling.

### Environment

A total of 43/236 (18.2%) environmental samples were MRSP-positive (Table 1). Positive environmental wipes were found in 68.8% (11/16), 28.6% (4/14), 0% (0/14), 30.8% (4/13), 0% (0/13) and 41.7% (5/12) of the households in the first to sixth sampling respectively. In general, MRSP-positive environmental wipes were found in combination with MRSP-positive animals. However, in four households MRSP-positive environmental wipes were found during a sampling without MRSP-positive animals (Table S1). The feeding place was MRSP-positive in 11 households, the sleeping place in nine and the site not physically accessible to the animal in six households.

### Genotyping results

In 12 households several MRSP isolates from different sampling times were available, in three households only isolates from the first sampling time were available, and in one household all samples were MRSP-negative. This resulted in a total of 60 isolates that were genotyped.

Genotype ST71-J-t02-II/III was the dominant type found in 8/16 (50%) households (Table 2). No ST71 strains were present in five households that instead harboured strains with ST29, 111, 115, 131 and 143, respectively. Also strains with different STs were found within two household (households 11 and 16) and strains that were non-typeable with PFGE using *Sma*I, but showed related banding patterns after digestion with *Cfr*9I, type Cfr1 and Cfr2, respectively (Table 2). Remarkable was the finding that *spa* typing further differentiated strains that were indistinguishable with MLST and PFGE. In three households, either *spa* types t02 and t05 (households 2 and 12) or *spa* types t02 and t06 (household 7) were found, although *spa* types (t02, t05 and t06) were considered to be closely related as they differed only in the total number of central r03 repeats (Table 2). SCC*mec* II/III was most frequently found and associated with isolates of ST71. SCC*me*c type V was found in combination with ST115. Isolates with ST29, 111 and 143 contained non-typeable SCC*mec* cassettes, as none of the multiplex PCR assays amplified a product (Table 2).

**Table 2:** Typing results of MRSP isolates.

Index	Sampling	Isolate from:	MLST	PFGE	Spa	SCCmec
**1**	1	index dog	71	J	t02	II/III
	4	environment	71	J	new	II/III
	6	index dog	71	J	t02	II/III
**2**	1	index dog	71	J	t02	II/III
	2	index dog	71	J	t05	II/III
**3**	1	index dog	29	Cfr1	t09	NT
	1	contact animal	29	Cfr1	t09	NT
	1	humans	29	Cfr1	t09	NT
	1	environment	29	Cfr1	t09	NT
	5	index dog	29	Cfr1	t09	NT
	6	contact animal	29	Cfr1	t09	NT
	6	environment	29	Cfr1	t09	NT
**4**	1	index dog	71	J	t02	II/III
	1	environment	71	J	t02	II/III
	2	index dog	71	J	t02	II/III
**5**	1	environment	131	J	no	NT
**6**	1	index dog	111	U	no	NT
	2	index dog	111	U	no	NT
	3	index dog	111	U	no	NT
	4	index dog	111	U	no	NT
	6	index dog	111	U	no	NT
**7**	1	index dog	71	J	t02	II/III
	1	environment	71	J	t02	II/III
	2	index dog	71	J	t02	II/III
	2	contact animal	71	J	t06	II/III
	6	index dog	71	J	t06	II/III
	6	environment	71	J	t02	II/III
**8**	1	index dog	115	Q	new	V
	1	contact animal	115	Q	new	V
	1	environment	115	Q	new	V
	6	index dog	115	Q	new	V
	6	contact animal	115	Q	new	V
	6	humans	115	Q	new	V
	6	environment	115	Q	new	V
**9**	1	index dog	71	J	t02	II/III
	1	environment	71	J	t02	II/III
	2	environment	71	J	t02	II/III
	4	index dog	71	J	t02	II/III
**10**	1	index dog	71	J	t02	II/III
	6	index dog	71	J	t02	II/III
**11**	1	index dog	71	J	t02	II/III
	1	contact animal	71	J	t02	II/III
	1	environment	29	Cfr2	t09	NT
**12**	1	index dog	71	Y	t02	II/III
	1	contact animal	71	Y	t05	II/III
	1	humans	71	Y	t02	II/III
	1	environment	71	Y	t02	II/III
**13**	1	index dog	143	G	no	NT
	1	humans	143	G	no	NT
	1	environment	143	G	no	NT
	6	index dog	143	G	no	NT
	6	environment	143	G	no	NT
**14**	1	index dog	71	J	t06	II/III
	1	environment	71	J	t06	II/III
	6	index dog	71	J	t06	II/III
	6	environment	71	J	t06	II/III
**15**	NA	NA	NA	NA	NA	NA
**16**	1	index dog	29	Cfr2	t09	NT
	1	contact animal	29	Cfr2	t09	NT
	1	environment	29	Cfr2	t09	NT
	2	environment	71	J	t02	II/III

NA: No MRSP-isolates available.

NT: non-typeable.

## Discussion

To our knowledge, this is the first study investigating the occurrence of MRSP within a household with a (former) canine MRSP patient in time. The sampling results of the sixteen different households showed considerable variation in the persistence of MRSP. Although two dogs were continuously MRSP-positive during six months, dogs could also be MRSP-positive intermittently, occasionally with up to three months between two MRSP-positive samplings.

On the one hand, dogs with clinical signs and a proven MRSP infection in the past were not always MRSP-positive. As selective culturing was used and different sites were sampled (nose and perineum), the possibility of a false-negative culture result was greatly reduced. On the other hand one dog was even MRSP-positive more than one year after the initial sampling showing that MRSP can persist in dogs. As this was a field study and dogs with different clinical conditions were included, different treatment regimens were applied to the index cases. This could have affected the presence of MRSP. Index cases, which became MRSP-negative, however, included both dogs with and without a treatment. The same MRSP genotype was found in dogs without clinical signs for several months, suggesting long-term colonization rather than transient colonization. Taken together, these results show that veterinarians should stay alert with (former) MRSP patients, even after repeated MRSP-negative cultures or after the disappearance of the clinical infection.

This field study was performed in a setting with MRSP patients from different veterinary clinics in the Netherlands. The clinical condition, household situation, and/or provided therapies could have contributed to the variation in the presence of MRSP. Moreover, the study was performed from March to October 2010, therefore potential seasonal influences, including allergen exposure could not be excluded.

In addition to external influences, animal specific factors could also have played a role in the prevalence and persistence of MRSP in some canine patients. With *S. aureus* several factors are known to influence the rate of nasal carriage in humans [25]. For *S. pseudintermedius,* studies on the risk factors for colonization are rare. The presence of skin lesions, previous hospitalization and previous antimicrobial therapy have been identified as a risk factors for carriage [2], [15], [26].

Animals in close contact with MRSP patients were frequently found MRSP positive, which was also described in a one point prevalence study by van Duijkeren et al.[27].

MRSP-positive contact animals were usually found in combination with MRSP-positive index dogs. However, in one household the index dog became MRSP-negative while the contact dog was repeatedly tested MRSP-positive with the same genotype that was originally isolated from the index case. During the study this contact dog received antimicrobials and was submitted to an animal hospital for health issues not related to MRSP. As MRSP are multidrug resistant this may have favoured colonization. Generally, contact animals carried the same MRSP-genotype as the index case. Only in two households (7 and 12) the contact animal carried MRSP with a different, but closely related, *spa* type. It shows that there is a high risk of transmission of MRSP to animals in close contact with MRSP patients and that veterinarians and owners should be aware of this risk.

In contrast to contact animals, humans are rarely found MRSP-positive [27], [28]. In this study five owners in four households were found MRSP-positive with four different sequence types (ST71, ST29, ST115, ST143). The MRSP-positive humans were found in combination with MRSP-positive index dogs showing clinical signs, contact animals and environmental samples indicating considerable exposure. After testing MRSP-positive, two of the five owners were tested repeatedly and they were not tested MRSP-positive more than once. Both owners were MRSP-positive with a rare genotype, namely ST29-*Cfr*1-t09-NT and ST143-G-no-NT respectively. No eradication therapy was performed. These results suggest occasional contamination or rapid elimination of colonization of the owners. However, in a recent study by Paul et al. [29] 5/128 small animal dermatologists were found MRSP-positive and two of them were re-tested one month later and both tested MRSP-positive again with an isolate with the same *spa*-type as in the initial screening. The authors suggest that MRSP with MLST ST71 and ST106 are more able to colonize humans. However, it is also possible that the veterinarians were re-infected as they have frequent contact with infected pets.

In the present study, the majority of MRSP-positive environmental samples were those in which there was physical contact with the index case, indicating that physical contact is an efficient way of MRSP-transmission. The study of van Duijkeren et al. [27] shows that the feeding and sleeping place are most often found MRSP-positive, which is in concordance with this study. In six households, however, MRSP was found at the site where no physical contact was possible with the index case or contact animal. In addition, physical contact of the owners with these sites was scarce, because of poor accessibility. Therefore potential transmission of MRSP from the owner's hands to these sites was unlikely. However, a considerable amount of dust was collected at these sites each month, which indicates that besides physical contact, dust particles play a role in the maintenance and distribution of MRSP.

The emergence of MRSP in Europe is thought to be mainly due to clonal spread of one major clonal lineage MLST ST71-*spa* t02-SCC*mec* II-III. An interesting finding from the present study was that several different MLST types were found (ST71, ST29, ST111, ST115, ST131 and ST143), although MLST ST71 predominated. In general, similar or indistinguishable MRSP isolates were found in patients, contact animals and environmental samples within the same household indicating transmission within the household. In three households containing MRSP strains with ST111, ST115 and ST143 the same strain was found during the first and sixth sampling and no other strains were found, showing an ongoing infection or re-infection of the index dog with the same MRSP strain for six months. The risk of re-infection with MRSP should be considered since studies on the survival of *S. aureus* in the environment have shown that the bacteria can survive for a considerable amount of time in dust and the same may hold true for MRSP [30]. Moreover, in four households MRSP-positive environment wipes were found while all animals and humans at that time were MRSP-negative. Occasionally different genotypes were found within one household and within one sampling. In three households (2, 7 and 12) isolates were found that only differed in *spa* type. The obtained *spa* types belonged to types t02, t05 and t06 that differed only in the presence or absence of a central r03 repeat, and may suggest modification of the *spa* repeats after introduction of MRSP to the household rather than independent acquisition of different MRSP types. An argument in favor of this theory is that all isolates within these 3 households shared the same PFGE pattern, SCC*mec* cassettes and MLST type. However, the presence of multiple MRSP strains in one household should also be considered, as shown in two households (11 and 16) that harbored MRSP isolates with different STs. Studies have shown that different MRSP strains can coexist in one animal [26].

In conclusion, dogs infected with MRSP can become colonized with MRSP and remain MRSP-positive for prolonged periods of time. In addition, dogs can test MRSP-positive after repeated MRSP-negative samplings or after the disappearance of the clinical infection. MRSP is easily transmitted to contact animals and the environment, which both are occasionally MRSP-positive without the presence of an MRSP-positive index dog. The contact animals and the environment might be reservoirs for recurrent MRSP infections in the index case or new MRSP infections in other animals. Long-term colonization of dogs was found, but transmission to humans was rare and humans were never found MRSP-positive more than once, suggesting contamination instead of colonization with MRSP.

## Supporting Information

Table S1 Results of samplings in the 16 households.
(DOC)
