# Supplementary material for: Longitudinal Study on Methicillin-Resistant Staphylococcus pseudintermedius in Households
Source: PLoS One. 2011 Nov 23;6(11):e27788. doi: 10.1371/journal.pone.0027788 (PMC3223215; doi:10.1371/journal.pone.0027788)
Supplement: Table S1 — Results of samplings in the 16 households. (DOC) [file pone.0027788.s001.doc]

**Table S1. Results of samplings in the 16 households**

|  |  |  |  |  | **Samplings** | | | | | |
| --- | --- | --- | --- | --- | --- | --- | --- | --- | --- | --- |
| **Index** | **Clinical condition** | **First MRSP+ sample** | **First sampling** | **Isolate from** | **1** | **2** | **3** | **4** | **5** | **6** |
| **1** | Otitis externa* | 14-9-2009 | 22-4-2010 | index dog | + N,I | + N,P | - | - | - | + |
|  |  |  |  | contact animal | No | No | No | No | No | No |
|  |  |  |  | humans | - | - | - | - | - | - |
|  |  |  |  | environment | - | - | - | + | - | - |
| **2** | Pyoderma* | 30-11-2009 | 30-3-2010 | index dog | + P | + P | - | - | - | - |
|  |  |  |  | contact animal | No | No | No | No | No | No |
|  |  |  |  | humans | - | - | - | - | - | - |
|  |  |  |  | environment | - | - | - | - | - | - |
| **3** | Otitis externa* | 13-11-2009 | 6-4-2010 | index dog | + N, I | + P | + I | + N | + N | - |
|  |  |  |  | contact animal | + N | + P | - | - | + N | + N,P |
|  |  |  |  | humans | + | - | - | - | - | - |
|  |  |  |  | environment | + | - | - | + | - | + |
| **4** | Postoperative wound infection | 2-11-2009 | 1-4-2010 | index dog | +^†^ I | +^†^ I | - | - | - | x |
|  |  |  |  | contact animal | - | - | - | - | - | x |
|  |  |  |  | humans | - | - | - | - | - | x |
|  |  |  |  | environment | + | - | - | - | - | x |
| **5** | Pyoderma* | 5-11-2009 | 19-3-2010 | index dog | - | - | - | - | - | - |
|  |  |  |  | contact animal | No | No | No | No | No | No |
|  |  |  |  | humans | - | - | - | - | - | - |
|  |  |  |  | environment | + | - | - | - | - | - |
| **6** | Pyoderma | 30-11-2009 | 19-3-2010 | index dog | + P | + P | + P | + P | - | +^†^ P |
|  |  |  |  | contact animal | No | No | No | No | No | No |
|  |  |  |  | humans | - | - | - | - | - | - |
|  |  |  |  | environment | - | - | - | - | - | - |
| **7** | Postoperative wound infection | 13-10-2009 | 11-3-2010 | index dog | + P | + P | - | - | - | + P |
|  |  |  |  | contact animal | - | + N | - | - | - | - |
|  |  |  |  | humans | - | - | - | - | - | - |
|  |  |  |  | environment | + | - | - | - | - | + |
| **8** | Otitis externa* | 3-11-2009 | 15-3-2010 | index dog | + N,P,I | + I | + N,I | + I | + I | + N,I |
|  |  |  |  | contact animal | + | - | - | - | - | + |
|  |  |  |  | humans | - | - | - | - | - | + |
|  |  |  |  | environment | + | - | - | - | - | + |
| **9** | Pyoderma* | 28-12-2009 | 18-3-2010 | index dog | + P | + N,P | + P | + P | - | - |
|  |  |  |  | contact animal | No | No | No | No | No | No |
|  |  |  |  | humans | - | - | - | - | - | - |
|  |  |  |  | environment | + | + | - | - | - | - |
| **10** | Otitis externa* | 11-1-2010 | 8-4-2010 | index dog | + I | + I | - | - | - | + P,I |
|  |  |  |  | contact animal | No | No | No | No | No | No |
|  |  |  |  | humans | - | - | - | - | - | - |
|  |  |  |  | environment | - | - | - | - | - | - |
| **11** | Postoperative wound infection | 7-12-2009 | 22-4-2010 | index dog | + N,I | x | x | x | x | x |
|  |  |  |  | contact animal | + N,P | x | x | x | x | x |
|  |  |  |  | humans | - | x | x | x | x | x |
|  |  |  |  | environment | + | x | x | x | x | x |
| **12** | Pyoderma | 8-9-2009 | 26-3-2010 | index dog | + N,P,I | x | x | x | x | x |
|  |  |  |  | contact animal | + P | x | x | x | x | x |
|  |  |  |  | humans | + | x | x | x | x | x |
|  |  |  |  | environment | + | x | x | x | x | x |
| **13** | Wound* | 3-9-2009 | 29-3-2010 | index dog | + I | - | + P | + P,I | + P | + I |
|  |  |  |  | contact animal | No | No | No | No | No | No |
|  |  |  |  | humans | + | - | - | - | - | - |
|  |  |  |  | environment | + | + | - | + | - | + |
| **14** | Rhinitis* | 24-9-2009 | 18-3-2010 | index dog | + P | + P | + P | + P | + P | + N,P |
|  |  |  |  | contact animal | No | No | No | No | No | No |
|  |  |  |  | humans | - | - | - | - | - | - |
|  |  |  |  | environment | + | + | - | + | - | + |
| **15** | Postoperative wound infection | 12-1-2010 | 11-3-2010 | index dog | - | - | - | x | x | x |
|  |  |  |  | contact animal | No | No | No | x | x | x |
|  |  |  |  | humans | - | - | - | x | x | x |
|  |  |  |  | environment | - | - | - | x | x | x |
| **16** | Otitis externa* | 7-10-2009 | 14-4-2010 | index dog | + N | - | - | - | - | - |
|  |  |  |  | contact animal | + I | - | - | - | - | - |
|  |  |  |  | humans | - | - | - | - | - | - |
|  |  |  |  | environment | + | + | - | - | - | - |

*: clinical infection during 6 months

†: clinical infection during the sampling

N: MRSP-positive nasal swab

P: MRSP-positive perineal swab

I: MRSP-positive swab from the infection site

x: no samples taken
